# Morpho-molecular characterization reveals three *Cortinarius* subgenus *Dermocybe* species from southwest China (Agaricales, Cortinariaceae)

**DOI:** 10.3897/mycokeys.130.183563

**Published:** 2026-03-24

**Authors:** Yue Zhou, Liheng Mu, Qi Zhao, Yongsi Dai, Siqi Chen, Ting Wu, Ying Zhang

**Affiliations:** 1 Key Laboratory of Forest Disaster Warning and Control in Yunnan, College of Forestry, Southwest Forestry University, Kunming, Yunnan 650224, China Yunnan University Kunming China; 2 State Key Laboratory of Phytochemistry and Natural Medicines, Kunming Institute of Botany, Chinese Academy of Sciences, Kunming 650201, China Kunming Institute of Botany, Chinese Academy of Sciences Kunming China; 3 State Key Laboratory for Conservation and Utilization of Bio-Resources in Yunnan, Yunnan University, Kunming, China Southwest Forestry University Kunming China

**Keywords:** Cortinariaceae, morphology, new taxa, taxonomy

## Abstract

*Cortinarius* stands as the most extensive and morphologically distinctive genus within the Cortinariaceae. During our comprehensive surveys of macrofungi in southwestern China, we have collected mushroom specimens resembling *Cortinarius* and identified them as three new species: *Cortinarius orienticroceus*, *C. orientisanguineus*, and *C. subolivaceoluteus*, based on morphology and multi-locus phylogenetic analyses using maximum likelihood/Bayesian inference. *Cortinarius orienticroceus* is notable for its pileus, which is dark brown at the center and gradually lightens to a pale yellow at the edges; lamellae range from pale yellow to bright yellow, while the stalk is initially silky and shiny, later turning darker with a slight olive tint as it matures. *Cortinarius orientisanguineus*, on the other hand, features a dry, hemispherical pileus with noticeable fibrous scales, lamellae are reddish-brown, and the veil disappears over time, leaving a reddish-brown ring on the upper to middle part of the stalk. In contrast, *Cortinarius subolivaceoluteus* presents a pileus that starts papillate and flattens as it matures; the stalk is cinnamon-colored on the upper part, with tawny fibrils and patches below, and has a slightly bulbous base; the veil is cinnamon-colored and vanishes over time. Morphological illustrations and comprehensive morphological descriptions, along with phylogenetic relationships of other species within subg. *Dermocybe* of the three new *Cortinarius* species are provided. This research expands the taxonomic framework of subgenus *Dermocybe* through contemporary systematic approaches and emphasizes the substantial contribution of Chinese mycological collections to our understanding of this fungal group.

## Introduction

*Cortinarius* (Pers.) Gray is a genus classified within the class Agaricomycetes, order Agaricales, and family Cortinariaceae. This cosmopolitan genus demonstrates exceptional taxonomic diversity, encompassing more than 3,000 described species with a global distribution spanning all continents except Antarctica (Moser et al. 1975; Jacquemyn et al. 2010; Tedersoo et al. 2011; Harrower et al. 2015; Niskanen et al. 2018; Thoen et al. 2019; Li et al. 2025). *Cortinarius* is an ecologically important group of ectomycorrhizal fungi that establishes mutualistic symbioses with diverse plant taxa across various ecosystems. Some species, such as *Cortinarius armillatus* (Fr.) Fr., *C. bovinus* Fr., and *C. emodensis* Berk. possess both edible and medicinal value (Li et al. 2025).

Moser et al. (1983) proposed a taxonomic framework dividing the genus *Cortinarius* into six distinct subgenera: *Cortinarius*, *Leprocybe*, *Myxacium*, *Phlegmacium*, *Sericeocybe*, and *Telamonia*, while treating *Dermocybe* as an independent genus. Subsequently, Brandrud et al. (1998) refined this classification by consolidating the subgeneric structure into four subgenera: *Cortinarius*, *Myxacium*, *Phlegmacium*, and *Telamonia*, and incorporated *Dermocybe* within the subgenus *Cortinarius*. Later, Bidaud et al. (1994) established a modified system recognizing six subgenera: *Cortinarius*, *Dermocybe*, *Myxacium*, *Phlegmacium*, *Telamonia*, and *Hydrocybe*. The present study adopts the subgeneric classification proposed by Bidaud et al. (1994) with minor taxonomic modifications.

*Dermocybe* was originally distinguished from other subgenera of *Cortinarius* primarily on the basis of the presence of anthraquinone pigments, with only a few exceptions (Gruber 1970; Høiland and Holst-Jensen 2000). Nevertheless, its taxonomic status has been subject to ongoing debate (Moser 1978; Høiland 1983; Liu et al. 1997; Kuhnert-Finkernagel and Peintner 2003). *Dermocybe* sensu lato, hereafter referred to as dermocyboid species, comprises agaricoid fungi that are small to medium-sized and exhibit bright coloration ranging from yellow and red to brownish and olive hues. These species are characterized by a dry stipe and a pileus that varies from dry to slightly viscid, with a silky or occasionally waxy surface texture; the pileipellis displays a somewhat duplex structure with a poorly developed hypodermal layer, and the lamellae are typically adnate or adnexed, displaying yellow, red, orange, or green pigmentation. The partial veil is generally inconspicuous, while a cortina is consistently present; the odor is frequently indistinct, though some species may exhibit a radish-like scent (Liu et al. 1997; Knudsen 2008; Stefani et al. 2014; Soop 2021; Liimatainen et al. 2022; Xie et al. 2025a).

In the recent years, species of *Cortinarius* have been receiving much attention from mycologists, and many species were discovered from China (Wei and Yao 2013; Xie et al. 2019, 2020, 2021a, b, 2022, 2023a, b, 2024, 2025a, b; Yuan et al. 2020; Luo and Bau 2021; Xie 2022; Liu et al. 2023; Zhang et al. 2023; Zhou et al. 2023; Fan et al. 2024; Hong et al. 2024; Long et al. 2024; Dang et al. 2025; Guan et al. 2025; Jia et al. 2025; Wang et al. 2025a, b; Yang et al. 2025). In this study, a phylogenetic investigation of *Cortinarius* subg. *Dermocybe* species was identified using both morphological and molecular data (ITS+LSU). We attempt to 1) elucidate the species diversity of *Cortinarius* subg. *Dermocybe* in southwest China; 2) evaluate the phylogenetic relationships within the subgenus.

## Materials and methods

### Sample collection and morphological studies

From August 2014 to August 2023, seven distinct fungal collections were sourced from multiple localities across Yunnan Province, China, specifically including Lijiang, Honghe, Chuxiong, and Dali. Detailed metadata documentation was meticulously recorded for all collections following established taxonomic protocols (Rathnayaka et al. 2025). Macroscopic characteristics of the pileus, lamellae, and stipe for each collection were documented and photographed in situ. After being brought to the laboratory, the specimens were subsequently desiccated in a mushroom drier. Subsequent macroscopic morphological examination and microscopic photomicrography were conducted in the laboratory following the methodology of Zhao et al. (2015), with necessary modifications applied. The color codes referenced in the descriptions are based on Kornerup and Wanscher (1967), measurements are presented as (a–) b–c (–d), where a and d represent the absolute minimum and maximum values, and the range b–c encompasses at least 90% of all measurements. The notation [n/m/p] denotes the measurement of (n) basidiospores from (p) localities collected at (m) basidiomata. Furthermore, Q refers to the length-to-width ratio (aspect ratio) of the basidiospores, with values expressed as the mean (Q) ± standard deviation (SD). The newly collected specimens have been deposited in the Cryptogamic Herbarium of the Kunming Institute of Botany (herbarium code: **KUN-HKAS**).

### DNA extraction, PCR amplification, and sequencing

Genomic DNA was extracted from dried basidiocarps using the “trilief” Plant Genomic DNA Kit (Tsingke Biotechnology Co., Ltd., Beijing, China). The polymerase chain reaction (PCR) was used to amplify the ribosomal internal transcribed spacer (ITS) and the large subunit of the ribosomal RNA (LSU), with primers ITS1-F/ITS4 (White et al. 1990; Gardes and Bruns 1993), and LR0R/LR5 (Vilgalys and Hester 1990; Rehner and Samuels 1994), respectively. PCR amplification was performed in a total volume of 25 μL, including 21 μL Taq PCR Master Mix (TSINGKE TSE101, Tsingke Biotechnology Co., Ltd., Beijing, China), 1 μL forward primer, 1 μL reverse primer, and 2 μL DNA template. PCR conditions: Initial denaturation at 98 °C for 5 min; followed by 35 cycles of 98 °C for 30 sec (denaturation), 53 °C for 20 sec (annealing), and 72 °C for 30 sec (extension); with a final extension at 72 °C for 10 min. PCR products were verified by 1% ethidium bromide-stained agarose gel electrophoresis, and successful amplicons were sent for sequencing at Tsingke Biotechnology Co., Ltd. (Beijing, China).

### Sequence alignment and phylogenetic analyses

The newly generated sequences were carefully inspected and assembled using DNAMAN 9.0. Sequencing data from the recently collected specimens were integrated with relevant sequence data from the literature for comprehensive analysis (Huymann et al. 2024). Reference sequences of related taxa were retrieved from the NCBI database (https://www.ncbi.nlm.nih.gov/) to facilitate phylogenetic tree construction (Table 1). Multiple sequence alignment was performed using MAFFT v.7 (Katoh et al. 2019; Katoh and Standley 2013; available at: https://mafft.cbrc.jp/alignment/server/index.html). *Phlegmacium boreicyanites* and *P. cyanites* were designated as the outgroup taxa. Sequences were manually edited using BioEdit 7.0.9 (Hall 1999). The trimmed sequences were subsequently concatenated into a combined dataset using Sequence Matrix V.1.8 (Vaidya et al. 2011). The final dataset was analyzed using both Maximum Likelihood (ML) and Bayesian Inference (BI) methods. Maximum Likelihood (ML) analysis was conducted using IQ-TREE (http://iqtree.cibiv.univie.ac.at/, Trifinopoulos et al. 2016). The best-fit substitution model for each gene partition was selected using MrModeltest v.2.3 (Nylander et al. 2004). Phylogenetic analyses for all gene regions were performed under the ML criterion, with 1,000 bootstrap replicates. The best-fit substitution model for the ITS region was HKY+F+I+G4, while that for the LSU region was TN+F+I. Bayesian Inference (BI) was performed using MrBayes v3.2.6 (Ronquist et al. 2012). Four simultaneous Markov Chain Monte Carlo (MCMC) runs were performed for 2,750,000 generations, with trees sampled every 1,000 generations until the average standard deviation of split frequencies fell below 0.01. The first 25% of generations were discarded as burn-in to calculate posterior probabilities (PP) (Huelsenbeck and Ronquist 2001). The resulting phylogenetic trees were visualized using FigTree v.1.4.4 and further refined in Adobe Illustrator 2020 to optimize graphical presentation.

**Table 1.** Species names, collection localities, references, and corresponding GenBank accession numbers of the taxa used in the phylogenetic analysis in this study. New species described in this study are shown in red; holotype specimens are indicated in bold with a "T" suffix; and "–" signifies unavailability in GenBank.

Species	Voucher/Isolate no.	GenBank accession no.		Distribution	References
		ITS	LSU		
*Cortinarius neosanguineus* T	H:TN09-130	NR_120149	NG_067565	USA	Niskanen et al. (2013)
*C. rubrosanguineus*	G00110215 (G)	JN114091	–	France	Niskanen et al. (2013)
*C. sanguineus*	CFP737 (S)	JN114101	MK277655	Sweden	Niskanen et al. (2013)
*C. subsanguineus*	HMJAU48961	OP620653	OP620664	China	unpublish
*C. croceus* **T**	H:6031266	NR_131863	–	Sweden	Niskanen et al. (2014)
*C. cinnamomeus*	HMJAU44396	PQ443967	PQ489264	China	Xie et al. (2025a)
*C. uliginosus*	TUB011823	AY669584	AY669584	Germany	Garnica et al. (2005)
*C. rubrophyllus*	IBF20190002b	MZ357345	–	Austria	unpublish
*C. xiaojinensis*	HMJAU58895	OP620654	OP620665	China	Xie et al. (2025a)
*C. austrosanguineus*	MEL2089685	GQ890317	JX544895	Australia	Danks et al. (2010)
*C. olivaceoluteus*	HMJAU63751	OR364554	–	China	unpublish
*C. olivaceoluteus* **T**	WTU:J. F. Ammirati 8443	NR_131862	–	USA	Niskanen et al. (2014)
*C. indotatus*	CO1624	KJ421110	KJ421110	Germany	unpublish
*C. leptospermorum* **T**	PDD:27183	GU233325	GU233395	New Zealand	unpublish
***C. orienticroceus* T**	**HKAS 151551**	**PX624098**	**PX624114**	**China**	**This study**
***C. orienticroceus***	**HKAS 151552**	**PX624099**	**PX624115**	**China**	**This study**
*C. aurantioferreus*	KS CO1197	KJ421107	–	Germany	Huymann et al. (2024)
*C. mycenarum*	PDD:112369	MW263787	–	New Zealand	Huymann et al. (2024)
*C. sommerfeltii*	HMJAU44457	OP620652	OP620663	China	Xie et al. (2025a)
*C. salignus* **T**	IBF19760208	OL712388	–	Sweden	Huymann et al. (2024)
*Dermocybe cinnamomeolutea*	personal:N. Dam:ND14060	PX221272	–	Netherlands	unpublish
*D. cinnamomeolutea*	personal:I. Somhorst:IS19182	PX221162	–	Netherlands	Huymann et al. (2024)
*C. ferruginosus* **T**	MICH:11058	NR_153037	–	USA	Liimatainen et al. (2014)
*C. uliginosus*	personal:I. Somhorst:IS19190	PX221169	–	Netherlands	unpublish
*C. cinnamomeus* **T**	S:F44851	NR_131816	–	Sweden	Niskanen et al. (2013)
*C. holoxanthus* **T**	IBF19650150	OL712385	–	Austria	Huymann et al. (2024)
*C. ammiratii* **T**	H:T. Niskanen 09-146	NR_131860	–	USA	Niskanen et al. (2014)
*C. pitkinensis* **T**	WTU:F-040015	NR_131858	–	USA	Niskanen et al. (2014)
*C. subrufulus* **T**	H:T. Niskanen 09-207	NR_131853	–	USA	Niskanen et al. (2014)
*C. hadrocroceus* **T**	H:T. Niskanen 10-122	NR_131854	–	Canada	Niskanen et al. (2014)
*C. cascadensis* **T**	MICH:10330	NR_120147	–	USA	Niskanen et al. (2013)
*C. croceoconus* **T**	S:F44854	NR_131869	–	Germany	Niskanen et al. (2014)
*C. viridiflavus* **T**	WTU:J. F. Ammirati 11673	NR_131856	–	USA	Niskanen et al. (2014)
*C. bataillei*	MQ23-HRL3889	PP464335	–	Canada	unpublish
*C. bataillei*	YSU-F-12164	OP866252	–	Russia	Filippova et al. (2023)
*C. tillamookensis* **T**	MICH:139446	NR_131857	–	USA	Niskanen et al. (2014)
*C. transatlanticus* **T**	WTU:J. F. Ammirati 11244	NR_131855	–	USA	Niskanen et al. (2014)
*C. aurantiobasis* **T**	MICH:10318	NR_120146	–	USA	Niskanen et al. (2013)
***C. orientisanguineus***	**HKAS 151550**	**PX624096**	**PX624113**	**China**	**This study**
***C. orientisanguineus***	**HKAS 151549**	**PX624097**	**–**	**China**	**This study**
***C. orientisanguineus* T**	**HKAS 151548**	**PX624095**	**PX624112**	**China**	**This study**
*C. huronensis*	MICH:Ammirati 5403	PP001387	–	USA	Huymann et al. (2024)
*C. huronensis*	L:L.4338917	PX245958	–	Netherlands	Huymann et al. (2024)
*C. tubarius*	MICH:Smith 64673	PP001390	–	USA	Huymann et al. (2024)
*D. sphagnogena*	IBF19991068 _TG1999415	OQ549948	–	Austria	unpublish
*D. sphagnogena*	IBF19700271	OL712387	–	Denmark	unpublish
*C. trappei* **T**	WTU:J.F. Ammirati 10001	NR_131890	–	USA	Liu Y. (1995)
*C. semisanguineus*	CFP333 (S)	JN114090	–	Sweden	Niskanen et al. (2013)
*C. humboldtensis* **T**	MICH:10362	NR_120303	–	USA	Niskanen et al. (2013)
*C. semisanguineus*	DAVFP 29118	EU821654	–	Canada	Harrower et al. (2011)
*C. ominosus*	G:435756	PP001388	–	France	Huymann et al. (2024)
*C. ominosus*	DPL13331	PX270872	–	USA	unpublish
*C. marylandensis* **T**	WTU:F-040674	NR_120151	–	USA	Niskanen et al. (2013)
*C. idahoensis* **T**	MICH:5524	NR_120145	–	USA	Niskanen et al. (2013)
*C. chrysolitus* **T**	MICH:10332	NR_120302	–	USA	Niskanen et al. (2013)
*C. pellstonianus*	WTU-F-078544	PV083504	–	USA	unpublish
*C. pellstonianus*	LP_14	PQ436998	–	Canada	Huymann et al. (2024)
*C. sommerfeltii*	CFP594 (S)	JN114083	–	Sweden	Niskanen et al. (2013)
*C. appalachiensis* **T**	TENN:061675	NR_131852	–	USA	Niskanen et al. (2014)
*C. ceskae* **T**	UBC:F16374	NR_131806	–	Canada	Harrower et al. (2011)
*C. scruentiphyllus*	H:T. Niskanen 05-001	KP087972	–	Finland	Niskanen et al. (2014)
***C. subolivaceoluteus* T**	**HKAS 151553**	**PX624100**	**PX624116**	**China**	**This study**
***C. subolivaceoluteus***	**HKAS 151554**	**PX624101**	**–**	**China**	**This study**
*C. cruentiphyllus* **T**	H:6031523	NR_131848	–	Finland	Niskanen et al. (2014)
*C. cistoadelphus* **T**	IBF19940606	OL712389	–	Spain	Huymann et al. (2024)
*C. phoeniceus* **T**	S:CFP742	NR_157868	–	Sweden	Niskanen et al. (2013)
*C. phoeniceus*	HKAS 124757	PV715487	–	China	unpublish
*C. cruentus*	G00110213 (G)	JN114092	–	France	Niskanen et al. (2013)
*C. puniceus* **T**	K:PDO21091957	NR_119965	–	UK	Niskanen et al. (2013)
*C. sanguineus* **T**	UPS:SL22091940	NR_119967	–	Sweden	Niskanen et al. (2013)
*C. birkebakii* **T**	WTU:J. M. Birkebak 10-20-2007-18	NR_131849	–	USA	Niskanen et al. (2014)
*C. timiskamingensis* **T**	NBM:D. Malloch 3-9-81/2	NR_131850	–	Canada	Niskanen et al. (2014)
*C. pseudofervidus* **T**	S:F44496	NR_131817	–	Sweden	Niskanen et al. (2013)
*C. vitiosus* **T**	IB:MM1974/0117	NR_119966	–	Sweden	Niskanen et al. (2013)
*D. malicoria*	IBF20060552_TG2006048	OQ549955	–	Italy	unpublish
*D. malicoria*	IBF20200071	OL712404	–	Austria	Huymann et al. (2024)
*D. malicoria*	TN04-406	JX045668	–	Finland	Niskanen et al. (2013)
*C. rubrophyllus* **T**	G:R. Baubet No. 234	NR_153036	–	France	Niskanen et al. (2014)
*C. cruentoides* **T**	PDD:101864	NR_156275	–	New Zealand	unpublish
*C. cruentoides*	PDD:106892	MW263736	–	New Zealand	Huymann et al. (2024)
*C. mycenarum* **T**	PDD:107715	NR_156300	–	New Zealand	Huymann et al. (2024)
*C. elaiops* **T**	PDD:88271	NR_157903	–	New Zealand	Huymann et al. (2024)
*C. alienatus* **T**	PDD:27180	NR_119777	–	New Zealand	Huymann et al. (2024)
*C. uliginosus*	CFP1049 (S)	JN114081	–	Sweden	Niskanen et al. (2013)
*C. phoeniceus*	MQ24-HRL4325	PP763010	–	Canada	Huymann et al. (2024)
*C. uliginosus*	CFP1049 (S)	JN114081	–	Sweden	Niskanen et al. (2013)
*C. phoeniceus*	MQ24-HRL4325	PP763010	—	Canada	Huymann et al. (2024)
*Phlegmacium boreicyanites*	HMJAU44340	OM001482	OM001522	China	Xie et al. (2025a)
*P. cyanites*	HMJAU48681	MT299957	OM001523	China	Xie et al. (2025a)

## Results

### Phylogenetic analyses

**Figure 1.**
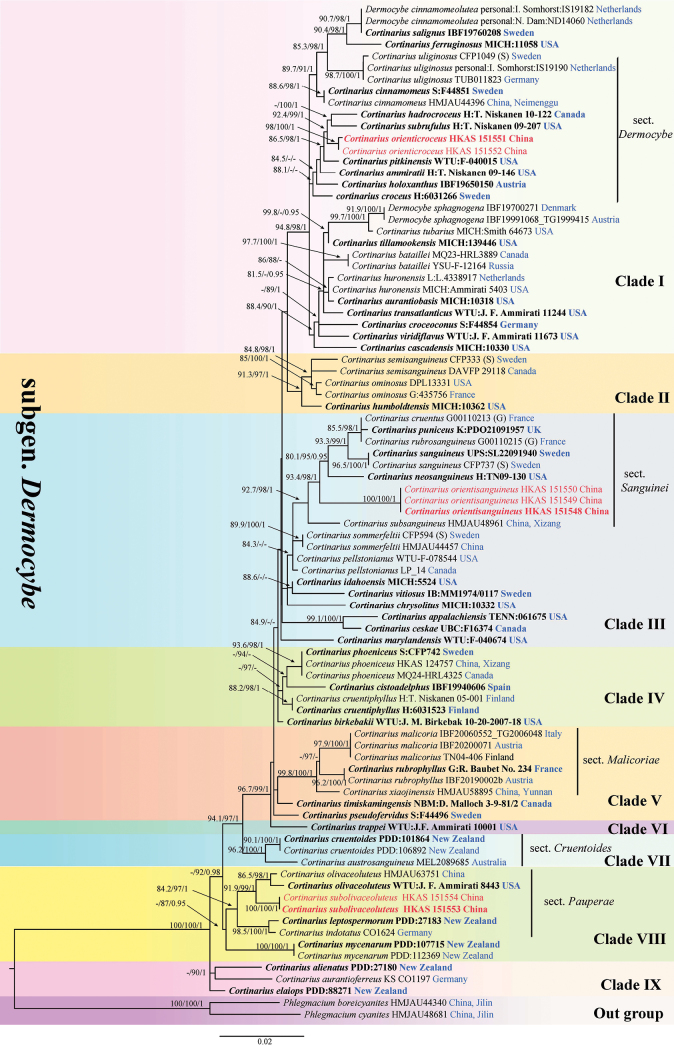
Phylogenetic tree inferred from the combined ITS and LSU sequence dataset using Maximum Likelihood (ML). Branch support values are indicated as “–/–/–” on the nodes (representing SH-aLRT/UFB/BIPP, respectively), with support thresholds considered significant at SH-aLRT ≥ 80%, UFB ≥ 90%, and PP ≥ 0.95. In this study, newly described specimens are highlighted in red, type specimens are indicated in bold, and collection localities are marked in blue.

The concatenated ITS-LSU dataset comprised 87 taxa with 1,560 aligned characters (ITS: 1-670 bp; LSU: 671-1,560 bp). Following the methodology established by Huymann et al. (2024) and Xie et al. (2025a), *Phlegmacium boreicyanites* and *P. cyanites* were selected as outgroups. The phylogenetic tree generated by Maximum Likelihood (ML) analysis was highly congruent with the topology obtained from Bayesian Inference (BI). Therefore, the ML tree topology is presented along with statistical values generated by ML bootstrap support (MLBS) and Bayesian posterior probabilities (BIPP) (Fig. 1).

In our phylogenetic study, all seven specimens of the three newly identified species were reliably grouped into three distinct, well-supported clades. Specifically, *C. orienticroceus* in section *Dermocybe*, *C. orientisanguineus* in sect. *Sanguinei*, and *C. subolivaceoluteus* in sect. *Pauperae*. Additionally, the branching into their respective sister clades was highly supported.

### Taxonomy

#### Cortinarius orienticroceus

Taxon classificationFungiAgaricalesCortinariaceae

Y. Zhou, Y. Zhang & Q. Zhao sp. nov.

28A621EA-6E93-5B33-B861-C8E7FA6935A8

Index Fungorum: IF904774

**Figure 2.**
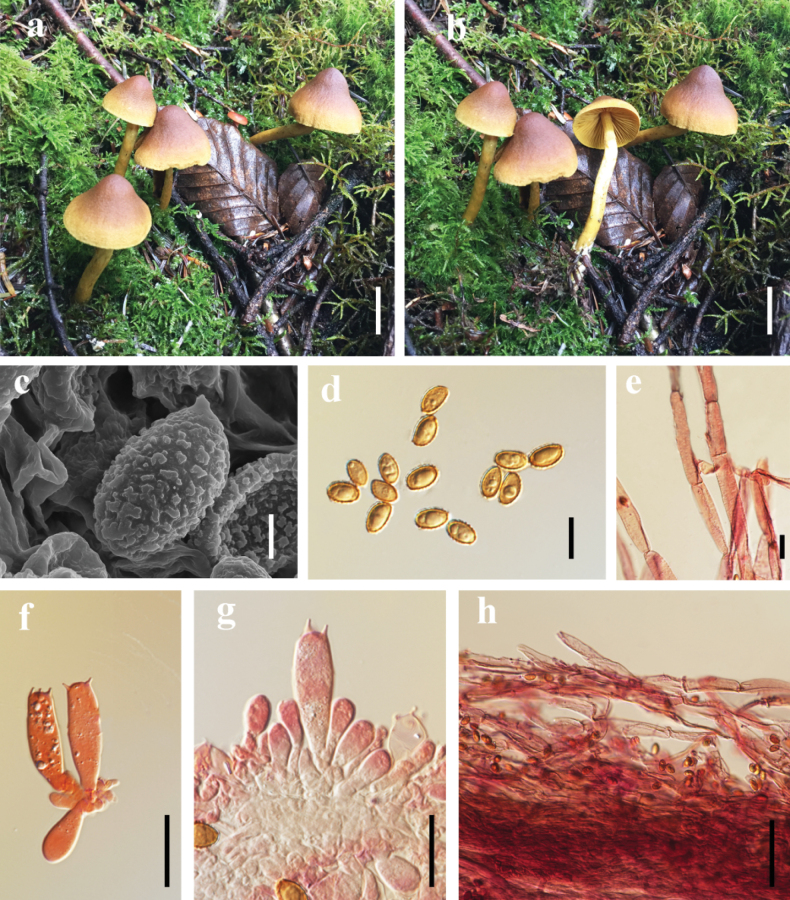
*Cortinarius orienticroceus*. **a, b**. Mature specimen (**a, b**. HKAS 151551 holotype); **c**. SEM micrographs of basidiospores; **d**. Basidiospores; **f, g**. Basidia; **e, h**. Pileipellis hyphae. Scale bar: 2 cm (**a, b**); 2 μm (**c**); 10 μm (**d**); 20 μm (**e–g**); 50 μm (**h**).

Fig. 2 Chinese name: 东方黄丝膜菌 (dong fang huang si mo jun)

##### Etymology.

Croceus means yellow, namely after its type locality from East Asia.

##### Holotype.

China, • Yunnan, Lijiang, Yulong Naxi Autonomous County, Jiuhe Township 26.630601°N, 99.722506°E; alt. 3236 m; in *Abies* forest; 28 Jul. 2016, LJ_343 (HKAS 151551).

##### Macrostructures.

The pileus measures 30–40 mm in diameter; it is campanulate, umbilicate, with a slightly incurved and non-striate margin; the surface is fibrillose-tomentose and non-viscid, appearing dark brown (9F6) at the disc and gradually fading to light brown (9E5) towards the margin, which is pale yellow (5D5). The lamellae are adnexed, moderately crowded, of unequal length, with even edges, and colored pale yellow to bright yellow (5D6). The stipe measures 50–80 mm long × 5–8 mm thick, cylindrical, and central. Its upper and median portions are pale yellow (5D6), while the base is reddish-brown (8B6). Initially silky and shiny, it darkens with maturity, developing slight olivaceous tones (dull pale yellow, 4D8). The veil is inconspicuous. The basal mycelium is light yellow (3A2). No distinct odor is detected from the fruiting body.

##### Microstructures.

Basidiospores [80/2/2] (6–) 6.5–8 (–8.5) × 4.5–6 (–6.5) μm,[Q = (1–) 1.21–1.68 (–2), **Q** = 1.48 ± 0.15], ellipsoid, moderately verrucose, amber-yellow. Basidia (22.5–) 23–32 × 6–9 μm, 4–spores, turning pale red to red in 10% KOH solution. Cystidia absent. Pileipellis duplex: the epicutis consisting of appressed hyphae 5–15 μm wide, cylindrical and elongated, subparallelly arranged on the epidermis, mostly hyaline and occasionally pale pink in 10% KOH, with clamp connections present; the subpellis not distinctly delimited from the context. Stipitipellis hyphae 7–18 µm wide, hyaline in 10% KOH, with clamp connections.

##### Habitat.

It forms ectomycorrhizal associations and grows gregariously in coniferous forests dominated by *Picea* spp. and *Abies* spp.

##### Distribution.

Located in northwestern Yunnan, China.

##### Additional material examined.

China, • Yunnan, Lijiang City, Laojunshan Nature Reserve, 26.630601°N, 99.722506°E; alt. 3846 m, in a forest of *Picea* spp. and *Abies* spp., 9 Aug. 2023, YYW_05 (HKAS 151552).

##### Notes.

The most distinctive feature of *Cortinarius orienticroceus* is its vibrant fruiting body coloration: the pileus transitions from dark brown at the disc to pale brown or light yellow towards the margin, the lamellae are pale yellow to bright yellow, the stipe is light yellow above and reddish brown below, the basidiospores are ellipsoid and ornamented with verrucose projections. Genetic studies show that *C. orienticroceus* is most closely related to *C. subrufulus* and *C. hadrocroceus* (Fig. 1). In comparison, *C. subrufulus* features a pileus that is brown to reddish-brown, often with a faint yellowish hue near the margin when young, lamellae are orange to red-orange, and its stipe is pale yellow. *C. hadrocroceus* has a hemispherical pileus, dark brown at the center and transitioning to brown or reddish-brown at the edges. Its lamellae are moderately spaced, starting as olive-yellow and turning pale olive-brown as they mature. The stipe is cylindrical or slightly bulbous at the base and pale yellow (Niskanen 2014).

#### Cortinarius orientisanguineus

Taxon classificationFungiAgaricalesCortinariaceae

Y. Zhou, Y. Zhang & Q. Zhao sp. nov.

4CE5DA59-214D-5BB0-8A89-1E2EC9FDEEB3

Index Fungorum: IF904775

**Figure 3.**
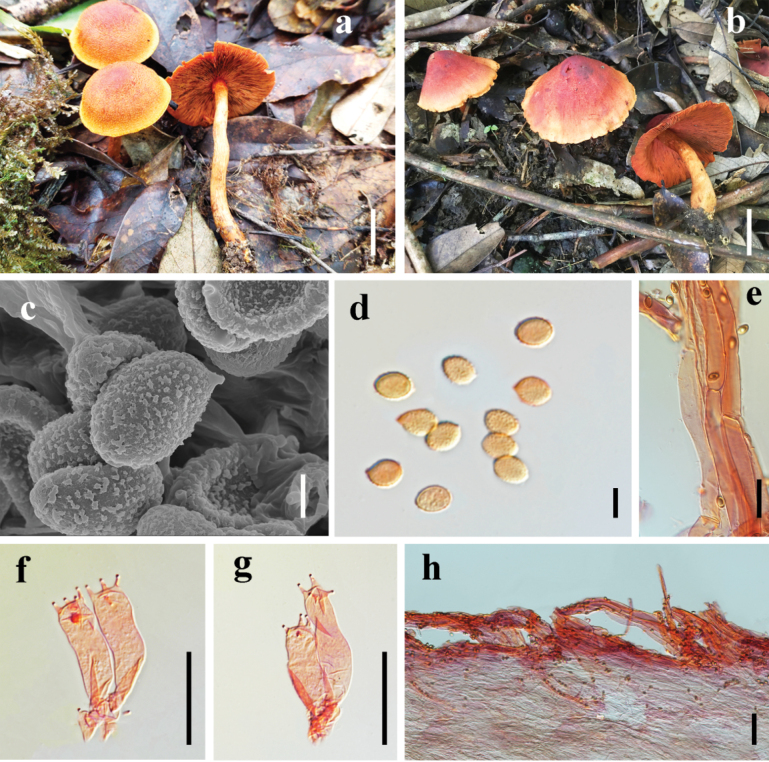
*Cortinarius orientisanguineus*. **a, b**. Mature specimen (**a**. HKAS 151549; **b**. HKAS 151548 **holotype**); **c**. SEM micrographs of basidiospores; **d**. Basidiospores; **f, g**. Basidia; **e, h**. Pileipellis hyphae. Scale bar: 2 cm (**a, b**); 2 μm (**c**); 5 μm (**d**); 20 μm (**e–g**); 50 μm (**h**).

Fig. 3 Chinese name: 东方血红丝膜菌 (dong fang xue hong si mo jun)

##### Etymology.

Sanguineus means blood-red, namely after its type locality from East Asia.

##### Holotype.

China • Yunnan, Honghe Hani and Yi Autonomous Prefecture, Pingbian Miao Autonomous County, Dawei Mountain National Forest Park, 22.5306201°N, 103.722405°E, alt. 2354 m, in evergreen broad-leaved forest, 14 Jul. 2020, PB_116 (HKAS 151548).

##### Macrostructures.

The pileus measures 28–42 mm in diameter, hemispherical, with an initially straight margin then becomes partly erose; the surface exhibits conspicuous fibrillose squamules and is dry. It is reddish-brown (9A8) at the disc, gradually fading to light brown (9A7) toward the margin, which is orange-yellow (5A6). The lamellae are crowded, of unequal length, adnate, and reddish-brown (8C7). The stipe measures 40–60 mm long and 3–4 mm thick, central, slightly bulbous at the base; its upper and median portions are reddish-brown (8C7), while the lower part is orange-yellow (6B6). The veil is evanescent, forming a reddish-brown annulus on the upper to median stipe. The basal mycelium is yellow (3A2).

##### Microstructures.

Basidiospores [120/3/1] (5–) 5.5–7.5 (–8) × 3.5–5.5 (–6) μm [Q = 1–1.5 (–1.65), **Q** = 1.39 ± 0.14], amygdaliform to ellipsoid, moderately verrucose, yellowish-brown. Basidia (18–)21–32 × 5–8 μm, 4–spores, containing granular contents, hyaline to pale pink in 10% KOH solution. Cystidia absent. Pileipellis exhibits a purplish-red coloration in KOH solution; epicutis well-developed, 8–15 μm wide, highly gelatinized, and the pileipellis exhibits elongate-ellipsoidal elements, closely interwoven and appressed to the epidermal layer, with clamp connections present, hyaline in 10% KOH, the hypodermal hyphae are inconspicuous. Stipitipellis hyphae 5–11 µm wide, hyaline in 10% KOH, with clamp connections.

##### Habitat.

It forms ectomycorrhizal associations and grows gregariously in evergreen broad-leaved forests.

##### Distribution.

It is situated in southern Yunnan, specifically in the southeastern part of the Honghe Hani and Yi Autonomous Prefecture.

##### Additional material examined.

China, • Yunnan, Honghe, Pingbian Miao Autonomous County, Dawei Mountain National Forest Park, 22.8616201°N, 103.523405°E, alt. 2341 m, in evergreen broad-leaved forest, 14 Jul. 2020, PB_68 (HKAS 151549), 15 Jul. 2020, PB_115 (HKAS 151550).

##### Notes.

*Cortinarius orientisanguineus* is distinguished by its pileus, which displays a reddish-brown center, transitioning to light brown towards the edges, and finally to orange-yellow at the margin. Its stipe is slightly enlarged at the base, with reddish-brown hues dominating the upper and middle sections, fading to orange-yellow near the base. The lamellae are closely spaced and uneven. The species also features moderately warted basidiospores, which are almond-shaped to elliptical, and basidia with granular contents. Phylogenetic studies indicate that *C. orientisanguineus* is closely related to the *C. sanguineus* complex, with the strongest similarity to *C. neosanguineus* (Fig. 1).

Morphologically, *C. neosanguineus* differs by having a uniformly bright red pileus, moderately spaced, deep purplish-red lamellae, and a stipe that matches the color of the lamellae (Niskanen et al. 2013). Additionally, the basidiospores of *C. neosanguineus* are larger, 7–8.5 (9) × 4.5–5. 5 μm. In comparison, *C. orientisanguineus* has smaller basidiospores, ranging from 5.5–7.5 × 4–5.5 (6) μm.

#### Cortinarius subolivaceoluteus

Taxon classificationFungiAgaricalesCortinariaceae

Y. Zhou, Y. Zhang & Q. Zhao sp. nov.

F13A19C1-0350-5972-BFB5-3C070D584806

Index Fungorum: IF904776

**Figure 4.**
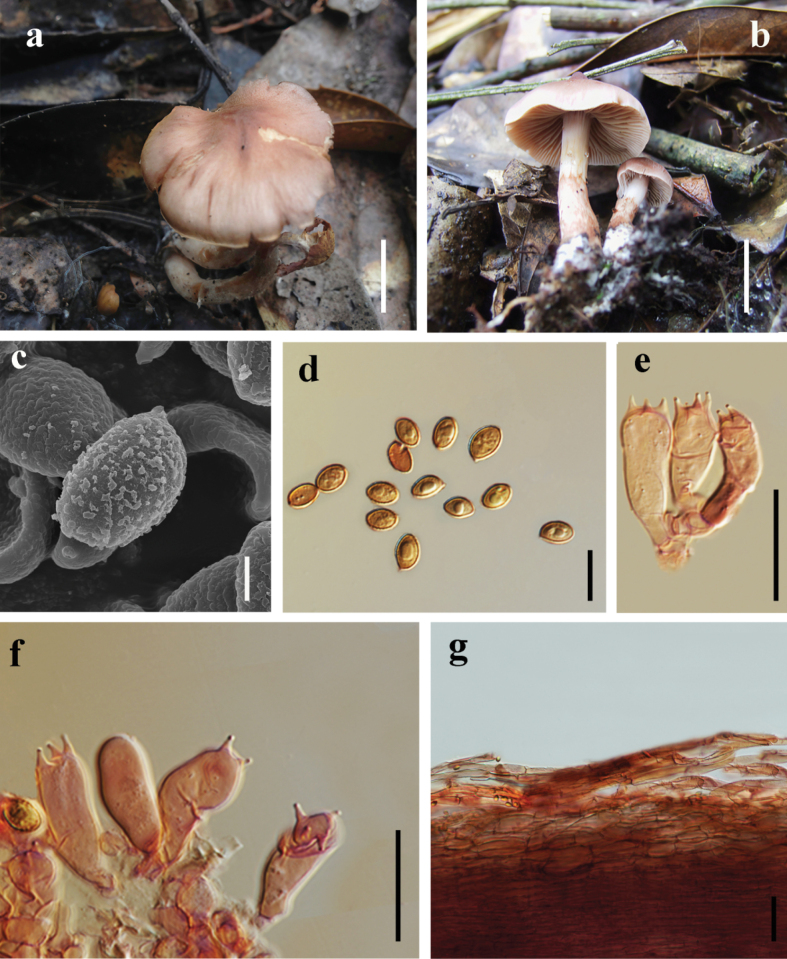
*Cortinarius subolivaceoluteus*. **a, b**. Mature specimen (**a**. HKAS 151553 **holotype**; **b**. HKAS 151554); **c**. SEM micrographs of basidiospores; **d**. Basidiospores; **e, f**. Basidia; **g**. Pileipellis hyphae. Scale bar: 2 cm (**a, b**); 2 μm (**c**); 10 μm (**d**); 20 μm (**e, f**); 50 μm (**h**).

Fig. 4 Chinese name: 拟橄榄绿丝膜菌 (ni gan lan lü si mo jun)

##### Etymology.

Olivaceoluteus means olive-green, named for the pileus color which resembles olive green but is distinctly different.

##### Holotype.

China, • Yunnan, Chuxiong, Zixishan Provincial Nature Reserve, 25.1301281°N, 101.221405°E; alt. 2502 m, in semi-humid evergreen broad-leaved forest, 18 Aug. 2014. ZXS_01_31 (HKAS 151553)

##### Macrostructures.

The pileus measures 20**–**40 mm in diameter, papillate at the disc when young and becoming applanate at maturity. The disc is tawny (7E7), gradually paling toward the margin, which is pale olivaceous or cinnamomeous (7D5), slightly incurved, and non-striate. The entire surface is fibrillose and dry. The lamellae are moderately crowded to moderately spaced, unequal in length, and cinnamomeous to pale olivaceous (7F8). The stipe is 30**–**45 mm long, cinnamomeous (7F8) on the upper and median parts, with reddish-brown (8D4) fibrils and patches below, and has a distinctly bulbous base. The veil is evanescent, cinnamomeous or pale olivaceous (7F8). The basal mycelium is pale grayish-white (7A1).

##### Microstructures.

Basidiospores [80/2/2] (5.64–) 6–7.5 × 3.5–4.5 (–5) μm [Q = 1–1.5 (–1.65), Q = 1.39 ± 0.16], ellipsoid, moderately verrucose, amber-yellow. Basidia 18–27 (–28) × 6–8 µm, 4–spores, hyaline to pale pink in 10% KOH solution. Pileipellis duplex: epicutis consisting of cylindrical to inflated, appressed hyphae 7–11 µm wide, hyaline in 10% KOH, the hypodermal hyphae 14.5–20 μm wide, broadly ellipsoid. Stipitipellis hyphae 10–14 µm wide, hyaline in 10% KOH, with clamp connections present.

##### Habitat.

It forms ectomycorrhizal associations and grows gregariously in semi-humid evergreen broad-leaved forests.

##### Distribution.

It is situated in the western part of Chuxiong City, Yunnan, China.

##### Additional material examined.

China, • Yunnan, Dali, Yongping County, Jinguangsi Provincial Nature Reserve, 25.0013201°N, 99.172805°E, alt. 2441 m, in evergreen broad-leaved forest, 7 Sep. 2009, JGS_22_35 (HKAS 151554).

##### Notes.

*Cortinarius subolivaceoluteus* is distinguished by its pileus which displays a tawny central disc that gradually transitions to a paler margin, exhibiting pale olivaceous to cinnamomeous coloration, the lamellae are cinnamomeous-colored, moderately spaced, and exhibit variable lengths, the stipe presents striking color variation: the upper and middle portions are cinnamomeous, while the lower section is decorated with reddish-brown fibrillose structures and patches, terminating in a prominently bulbous base. The species possesses an evanescent, pale olivaceous partial veil.

Phylogenetic analyses demonstrate that *C. subolivaceoluteus* forms a sister lineage to *C. olivaceoluteus* (Fig. 1). Morphologically, *C. olivaceoluteus* exhibits a uniformly cinnamomeous pileus ornamented with fine fibrillose squamules, densely arranged olive-yellow lamellae, and a clavate stipe that is concolorous with the lamellae and lacks surface squamulation (Niskanen 2014). Additionally, spore morphometry provides a reliable diagnostic character, *C. olivaceoluteus* basidiospores 7–8(–8.5) × 4–5(–5.5) μm, while *C. subolivaceoluteus* has smaller basidiospores6–7.5 × (3.5–)4–4.5(–5) μm.

## Discussion

China’s southwestern region harbors abundant fauna, flora, and fungal diversity. Over the past several decades, researchers have documented numerous novel fungal taxa from this biogeographically significant area (Yu et al. 2024; Dang et al. 2025; Wang et al. 2025; Xie et al. 2025a, b; Xu et al. 2025; Yang et al. 2025). However, the subgenus *Dermocybe* within the genus *Cortinarius* has received comparatively limited taxonomic attention, resulting in a substantial knowledge gap regarding the species diversity of *Cortinarius* subg. *Dermocybe* in this region. This study introduces three newly identified species within *Cortinarius* subg. *Dermocybe*: *C. orienticroceus*, *C. orientisanguineus*, and *C. subolivaceoluteus*. These discoveries are supported by a detailed morphological examination and robust molecular phylogenetic analyses. The identification of these species significantly expands the known diversity of the subgenus and offers valuable insights into the subtle differences and evolutionary relationships among its various groups.

Morphologically, *C. orienticroceus* stands out due to its strikingly multicolored pileus, which is dark brown at the center, transitioning to light brown and culminating in a pale yellow margin, paired with a notably long stipe. It closely resembles *C. subrufulus*, though the latter displays a more vibrantly colored pileus and narrower spores. In contrast, *C. orientisanguineus* is distinct with its reddish-brown center fading to light brown with an orange-yellow margin, densely spaced lamellae, and a stipe that changes color. These traits clearly differentiate it from the similar *C. sanguineus*, supported by a consistent set of features. Additionally, *C. orientisanguineus* has smaller spores and basidia compared to its nearest relative, *C. neosanguineus*. Meanwhile, although *C. subolivaceoluteus* shows brighter hues, it can be reliably separated from *C. olivaceoluteus* by the latter’s unique olive tones and larger spores. These observations align with the taxonomic framework proposed by Moser et al. (1983), emphasizing that consistent microscopic differences, such as spore size and surface patterns, are essential for distinguishing species within this group.

Currently, six recognized sections exist within the subg. *Dermocybe*: sect. *Dermocybe*, sect. *Aureifolii*, sect. *Cruentoides*, sect. *Malicoriae*, sect. *Pauperae*, and sect. *Sanguinei* (Liimatainen et al. 2022). Based on molecular phylogenetic analysis, the newly discovered species *Cortinarius orienticroceus*, *C. orientisanguineus*, and *C. subolivaceoluteus* belong to sect. *Dermocybe*, sect. *Sanguinei*, and sect. *Pauperae*, respectively. Species in subg. *Dermocybe* has considerable morphological variation in its fruiting bodies, ranging from small to medium-sized specimens. The pileus may be dry or moist, occasionally exhibiting hygrophanous properties or a sticky surface, with textures ranging from smooth to fibrillose or scaly. Molecular phylogenetic analyses have fundamentally transformed our understanding of this group’s taxonomic classification. Previously recognized as an independent genus based solely on morphological characteristics, subg. *Dermocybe* has been conclusively demonstrated, based on molecular evidence, to constitute a natural, monophyletic lineage within the genus *Cortinarius*, thereby confirming its evolutionary unity (Liu et al. 1997; Peintner et al. 2004; Garnica et al. 2005). Comprehensive molecular investigations of section *Sanguinei* have yielded particularly significant findings, not only validating its monophyletic status but also revealing substantially greater species diversity than morphology-based classifications had previously indicated (Niskanen et al. 2013). Conversely, section *Pauperae* exemplifies the numerous traditional morphology-based taxonomic groups within this subgenus that await molecular validation. The monophyletic nature and legitimate taxonomic standing of this section remain unresolved, underscoring the need for comprehensive type-specimen-based molecular phylogenetic research to reassess the many morphologically circumscribed groups within species-rich genera such as *Cortinarius* (Liimatainen 2013).

The taxonomic investigation of Subg. *Dermocybe* within China remains in its incipient phase (Yuan et al. 2020; Dang et al. 2025; Xie et al. 2025a), presenting substantial lacunae in our current understanding of this mycological assemblage. This nascent state of knowledge consequently presents considerable opportunities for advancing systematic mycology. Future investigations of *Dermocybe* should incorporate phylogenomic approaches to elucidate cryptic taxonomic diversity, thereby facilitating comprehensive systematic revision and enabling the description of novel taxa to augment the known biodiversity within this cortinarioid lineage.

## Supplementary Material

XML Treatment for Cortinarius orienticroceus

XML Treatment for Cortinarius orientisanguineus

XML Treatment for Cortinarius subolivaceoluteus

## Additional information

### Use of AI

No use of AI was reported.

### Author ORCIDs

Yue Zhou https://orcid.org/0009-0003-1972-2687

Liheng Mu https://orcid.org/0009-0002-6331-5010

Qi Zhao https://orcid.org/0000-0001-8169-0573

Yongsi Dai https://orcid.org/0009-0007-2143-5838

Siqi Chen https://orcid.org/0009-0003-5956-3123

Ting Wu https://orcid.org/0009-0003-5427-4808

Ying Zhang https://orcid.org/0009-0000-5248-0171

### Data availability

All of the data that support the findings of this study are available in the main text.
